# Exploring the Genetic and Morphological Basis of Biofilm-Linked Drug Resistance in Clinical Isolates of Acinetobacter baumannii

**DOI:** 10.7759/cureus.108958

**Published:** 2026-05-16

**Authors:** Upma Singh, Parul Jain, Atin Singhai, Rashmi, Sheetal Verma, RajKumar Kalyan, Vimala Venkatesh

**Affiliations:** 1 Microbiology, King George’s Medical University, Lucknow, IND; 2 Pathology, King George’s Medical University, Lucknow, IND

**Keywords:** bap gene, biofilm, blaoxa-23, csud gene, extensively drug-resistant (xdr), multidrug resistance (mdr), scanning electron microscopy (sem)

## Abstract

Background

*Acinetobacter baumannii* is a major nosocomial pathogen combining multidrug resistance (MDR) with strong biofilm-forming ability, enabling persistence on medical devices. Studying the correlation between biofilm-related genes and antibiotic resistance is critical for successful infection management. This study aimed to determine the prevalence of antibiotic resistance, phenotypic biofilm formation (by Congo red agar (CRA) and tissue culture plate (TCP) methods), and selected biofilm-associated genes (*bap*,* csuD*,* ompA*,* blaper-1*) in 153 clinical *A. baumannii* isolates from respiratory specimens, which were further characterized using scanning electron microscopy (SEM) analysis. In addition, the study aimed to evaluate the association between these genes, biofilm strength, and drug resistance patterns.

Methodology

In this study, an investigation was performed on 153 respiratory isolates from the laboratory of the Department of Microbiology at a tertiary care hospital (2022-2024). The Kirby-Bauer method was used for antibiotic susceptibility testing and minimum inhibitory concentration for colistin following the 2022 Clinical and Laboratory Standards Institute norms. Biofilm testing was performed using the CRA and TCP methods and validated by SEM and genetic analysis of biofilm association genes such as *bap*,* csuD*,* ompA*,* and blaPER-1.*

Results

The present study demonstrated an extremely high rate of antibiotic resistance, with meropenem resistance observed in 152 (99.3%) isolates and ceftriaxone resistance in 148 (96.7%) isolates. Regarding biofilm formation, the TCP method identified 85 (55.6%) isolates as biofilm producers, whereas the CRA method detected 57 (37.3%) isolates. Strong biofilm-producing isolates identified by the TCP method were confirmed by SEM to exhibit dense, mature biofilm structures. Genotypic analysis revealed *csuD (*99, 64.7%)and* bap (*82, 53.6%) as the most prevalent genes, both significantly associated with strong biofilm formation (p < 0.05). The combined presence of *bap *+* csuD* (51, 33.3%) showed a strong correlation with enhanced biofilm strength in the TCP assay (p < 0.05).

Conclusions

High MDR and strong biofilm formation were observed in *A. baumannii*, with a significant association between biofilm and antibiotic resistance. The* csuD* gene and *bap *+* csuD* combination correlated with increased resistance and biofilm production. SEM confirmed dense biofilm architecture, highlighting their role in virulence and persistence.

## Introduction

*Acinetobacter* is a Gram-negative coccobacillus, non-fastidious, non-fermenting, non-motile, oxidase-negative, catalase-positive, strictly aerobic bacterium that is an opportunistic pathogen. *Acinetobacter* species generally form smooth, greyish-white to pale yellow colonies on various bacteriological media [1]. The World Health Organization (WHO) has designated *A. baumannii* as a “critical priority” pathogen [2]. A recent Lancet report claimed an increased global burden of bacterial pathogens, which caused 4.95 million deaths in 2019. The Infectious Disease Society of America lists *A. baumannii* as one of the top priority antibiotic-resistant pathogens to be targeted because of its high potential to acquire drug resistance and the limited number of drugs available to treat illnesses caused by the bacteria [3,4]. Therefore, it is crucial to develop novel ways to tackle these antimicrobial-resistant bacterial pathogens with multidrug-resistant (MDR) and extremely drug-resistant (XDR) phenotypes reported in up to 90% of isolates in high-burden regions [5].

Biofilm formation plays a central role in the persistence and virulence of *A. baumannii*. Within these biofilms, bacteria are embedded in extracellular polymeric substances (EPS), which confer protection against antibiotics and host immune defences, enabling survival on abiotic surfaces such as endotracheal and tracheostomy tubes [6]. Device-associated infections linked to biofilm formation have been implicated in prolonged hospital stays, therapeutic failures, and increased morbidity [7]. MDR *A. baumannii* strains can become resistant to nearly all currently available antibiotics. In the most severe cases, isolates are susceptible only to polymyxins, such as colistin, leaving very limited treatment options [8]. Several mechanisms are responsible for this resistance. These include alterations in outer membrane proteins, decreased membrane permeability, frequent uptake of exogenous DNA, modification of penicillin-binding proteins, and activation of efflux pump systems. In addition, the bacterium can form biofilms and regulate gene expression through quorum sensing [9].

Hence, this study aimed to investigate (1) the antimicrobial resistance profile, (2) the phenotypic biofilm- forming ability using two methods (Congo red agar (CRA) and quantitative tissue culture plate (TCP) assay), and (3) the presence of four biofilm-related genes (*bap*,* csuD*,* ompA*,* blaPER-1*)by using conventional polymerase chain reaction (PCR) and scanning electron microscopy (SEM) in clinical *A. baumannii* isolates from respiratory samples. We also sought to examine correlations between these genes, biofilm production strength, and antibiotic resistance.

Knowledge gaps

Despite the clinical significance of *A. baumannii*, studies from India investigating antibiotic resistance patterns and the genetic mechanisms underlying antimicrobial resistance and biofilm formation, particularly those confirmed by SEM, remain limited. Additionally, to tackle the menace of biofilm formation, we aim to generate insights that may inform targeted infection-control strategies and therapeutic interventions.

## Materials and methods

This cross-sectional, laboratory-based study was conducted in the Department of Microbiology, King George’s Medical University (KGMU), Lucknow, India, from 2022 to 2024. A total of 153 isolates of *A. baumannii* were obtained from respiratory samples, including endotracheal aspirates, tracheostomy tubes, sputum samples, and branchial aspirates.

Ethical considerations

Ethical clearance for the study was obtained from the Institutional Ethics Committee (IEC) of KGMU, Lucknow (reference code 117th ECM II B-Ph.D./P4, dated June 3, 2022).

Sample size

The sample size for the present study was calculated using the standard formula for estimating proportions at a 95% confidence level, expressed as

\begin{equation} n = Z^2 \times p \times (1 - p) / d^2 \label{eq:placeholder_label} \end{equation}

where n represents the required sample size, Z is the standard normal deviate corresponding to the desired confidence level, p is the estimated prevalence, and d is the allowable error. Based on previously reported data [10], the prevalence of *A. baumannii* in respiratory samples was approximately 20%, with 50-70% of isolates reported as MDR.

Therefore, the expected prevalence of MDR *A. baumannii* was considered to be 10%. Using a confidence level of 95% (Z = 1.96) and an allowable error of 5%, the minimum sample size was calculated to be 138. To improve the reliability and validity of the study, a total of 153 isolates were included.

Inclusion and exclusion criteria

Clinical isolates of *A. baumannii *recovered from respiratory samples, including endotracheal aspirates, bronchoalveolar lavage fluid samples, sputum samples, and tracheal aspirates, were included in the study. Only non-duplicate isolates (one isolate per patient) were considered. Isolates obtained during the defined study period and confirmed by standard microbiological methods were considered.

Isolates obtained from non-respiratory specimens were excluded. Duplicate isolates from the same patient, contaminated cultures, and poor-quality samples were also excluded. Isolates with incomplete clinical or laboratory data, as well as those that could not be reliably identified as *A. baumannii*, were excluded from the study.

Characterization and identification of bacterial isolates

The identification of the isolates was performed using standard laboratory methods, including Gram stain, colony morphology, lactose fermentation, and oxidase test; biochemical tests such as methyl red-Voges-Proskauer test, triple sugar iron agar test, citrate, and oxidase test; and matrix-assisted laser desorption/ionization time-of-flight mass spectrometry (BioMérieux, Germany). MacConkey agar was used for obtaining pure culture colonies during growth at 37°C. A growth culture was obtained in Luria broth (LB) containing test tubes at 37°C under shaking. All strains were preserved in glycerol stock (LB containing 10% v/v glycerol) and kept at -80°C.

Antimicrobial susceptibility testing

According to the 2022 Clinical and Laboratory Standards Institute (CLSI) norms, the Kirby-Bauer diffusion method using disks was used for antimicrobial susceptibility testing (AST). Amikacin, gentamicin, tobramycin, cefepime, ceftriaxone, co-trimoxazole, piperacillin-tazobactam, levofloxacin, ciprofloxacin, imipenem, meropenem, and tetracycline were among the antibiotics tested (Hi-media, India) (Table 1), with quality control strains* *of *Escherichia coli* ATCC 25922 and *Pseudomonas aeruginosa* ATCC 27853.

**Table 1:** Zone diameter breakpoint of Acinetobacter baumannii.

Antimicrobial agent	Disc content	Zone diameter breakpoints (mm)		
		Sensitive	Intermediate	Resistant
Amikacin	30 μg	≥17	15–16	*≤*14
Cefepime	30 μg	≥18	15–17	*≤*14
Ceftazidime	30 μg	≥18	15–17	*≤*14
Ciprofloxacin	5 μg	≥21	16–20	*≤*15
Ceftriaxone	30 μg	≥21	14–20	*≤*13
Piperacillin-tazobactam	100/10 μg	≥21	18–20	*≤*17
Imipenem	10 μg	≥22	19–21	*≤*18
Meropenem	10 μg	≥18	15–17	*≤*14
Gentamicin	10 μg	≥15	13–14	*≤*12
Tobramycin	10 μg	≥15	13–14	*≤*12
Levofloxacin	5 μg	≥17	14–16	*≤*13
Tetracycline	30 μg	≥15	12–14	*≤*11

Isolates were classified as MDR if they exhibited resistance to at least one antibiotic in three or more antimicrobial categories, while XDR was defined as resistance to all antibiotics but one or two of the same or different categories.

Minimum inhibitory concentration for colistin susceptibility testing

The minimum inhibitory concentration (MIC) of colistin for *A. baumannii* isolates was determined using the broth microdilution method according to 2022 CLSI guidelines. Cation-adjusted Mueller-Hinton broth (CA-MHB) was used as the test medium. Colistin sulfate (HiMedia, India; potency 19,000 IU/mg) was prepared as a stock solution at a concentration of 1,024 µg/mL.

A final inoculum of approximately 5 × 10⁵ CFU/mL was achieved in each well by appropriate dilution of the bacterial suspension. Colistin was serially two-fold diluted in a 96-well microtiter plate to obtain final concentrations ranging from 0.12 µg/mL to 512 µg/mL.

Quality control strains included *E. coli* ATCC 25922 and *P. aeruginosa* ATCC 27853. CA-MHB without inoculum served as the negative control, while CA-MHB with bacterial inoculum served as the positive control. The plates were incubated aerobically at 37°C for 18-24 hours.

The MIC of colistin was defined as the lowest concentration at which the observed bacterial growth completely disappeared (no turbidity). Only clear wells were considered inhibitory; if a well was skipped (growth occurred at a higher concentration but not at a lower concentration), the test was redone. Colistin susceptibility was interpreted in accordance with the CLSI norms: susceptible at ≤2 µg/mL and resistant at ≥4 µg/mL.

Phenotypic and genotypic evaluation of biofilm formation

Congo Red Agar Method

CRA medium was prepared by supplementing 37 g/L of brain heart infusion agar with 0.8 g of Congo red dye and 36 g of sucrose. *A. baumannii* isolates were streaked onto the prepared plate and incubated at 37°C for 24 hours. Colony morphology was then assessed to determine biofilm production. Isolates that formed black colonies with dry crystalline consistency were considered strong biofilm producers, those with less intense black pigmentation were classified as weak biofilm producers, and isolates forming pink or red colonies were interpreted as non-biofilm producers.

Tissue Culture Plate Method

The quantitative evaluation of biofilm production was performed using the TCP method. Bacterial cells were cultured overnight at 37°C in tryptic soy broth (TSB) containing 0.25% glucose. A total of 180 µL of TSB and 20 µL of bacterial suspension (adjusted to a concentration of 0.5 McFarland) were added to a 96-well flat-bottomed polystyrene tissue culture plate in triplicate (three wells for each strain). A known biofilm-producing strain (*A. baumannii* ATCC 19606) served as a positive control, whereas sterile broth was used as a negative control. Following 24 hours of incubation at 37°C, the planktonic cells in each well were gently washed with phosphate-buffered saline (pH 7.2) to remove non-adherent cells and air-dried at 60°C for one hour. Adherent biofilm-forming cells were fixed in 200 µL of methanol for 15 minutes, stained with 0.2 mL or 2% crystal violet for 20 minutes, and then rinsed thoroughly with tap water to remove excess discoloration. The optical density (OD) of each well was then measured at 600 nm using a microplate spectrophotometer. Each test was performed in triplicate, and the average OD was noted.

DNA Extraction Method

The boiling method was used for the DNA extraction of isolates. For this, two to four well-isolated colonies were selected from the culture plate and inoculated into 200 µL of nuclease water in a sterile microcentrifuge tube. The suspension was mixed and vortexed for 20-30 seconds for complete homogenization. The tube was placed on a heating block for 10-15 minutes at 95°C-100°C for lysing the bacterial cell and releasing the genomic DNA. After heat lysis, the samples were centrifuged at 10,000 rpm for 10 minutes at 4°C. The pellet was discarded, and the clear supernatant containing DNA was carefully transferred into a sterile microcentrifuge tube. The extracted DNA was stored at -20°C until further use.

Genotypic Methods

For the detection of biofilm-related genes *bap*,* ompA*,* csuD*, and* blaPER-1*, a PCR assay was performed with genomic DNA in a 25 µL total reaction volume. Table 2 presents the biofilm gene sequence, base pair, and cycling temperatures. The PCR reaction mixture contained 12.5 µL of PCR master mix (Thermo-Fisher Scientific), 1 µL of each primer (10 pmol) (Eurofins genomics), 5.5 µL of nuclease-free water (Merck), and 5 µL of template DNA. A thermal cycler was used for amplification. A previously confirmed gene-positive *A. baumannii* isolate was used as a positive control, while nuclease-free water was used as a negative control for each PCR run.

**Table 2:** Primer sequence, amplicon size, cycling conditions, and references for polymerase chain reactions.

Target genes	Primer sequences (forward and reverse) (5’ to 3’)	Amplicon size (bp)	Cycling conditions
ompA	F- GTTAAAGGCGACGTAGACG R- CCAGTGTTATCTGTGTGACC	578	Initial denaturation: 15 minutes at 95°C; 35 cycles. Denaturation: 15 seconds at 95°C. Annealing: 60 seconds at 60°C. Extension: 1 minute for 72°C. Final extension: 1 minute at 72°C
bap	F-TGCTGACAGTGACGTAGAACCACA R-TGCAACTAGTGGAATAGCAGCCCA	184	Initial denaturation: 5 min at 94°C; 35 cycles. Denaturation: 50 seconds at 94°C. Annealing: 30 seconds at 55°C. Extension: 30 seconds at 72°C. Final extension: 5 minutes at 72°C
csuD	F-ATACCGACCTTTCACGGCTG R-GCCAGTATCGCCCTGCTTAT	335	Initial denaturation: 5 minutes at 94°C; 35 cycles. Denaturation: 50 seconds at 94°C. Annealing: 30 seconds at 55°C. Extension: 30 seconds at 72°C. Final extension: 5 minutes at 72°C
blaPER-1	F-GCTCCGATAATGAAAGCGT R-TTCGGCTTGACTCGGCTGA	520	Initial denaturation: 15 minutes at 95°C; 30 cycles. Denaturation: 30 seconds at 94°C. Annealing: 1.5 minutes at 59°C. Extension: 1.5 minutes at 72°C. Final extension: at 72°C for 10 minutes

Gel Electrophoresis

The PCR product was analyzed with 2% agarose gel electrophoresis, prepared with 1× TBE buffer (Thermo-Fisher Scientific, Invitrogen Bioservices, India) at room temperature. A total of 5 µL of PCR product of each isolate was added to a gel well along with 3 µL of DNA loading dye. A 100 bp and 150 bp DNA ladder (Thermo-Fisher Scientific, USA) was employed as a molecular weight marker. Electrophoresis was conducted at a constant voltage appropriate for the gel concentration until adequate separation was achieved. The gel was visualized for DNA bands under the analytical Gel Documentation System (Jena) with a CCD camera (NEXT-510, Analytical Jena, APH89).

Scanning electron microscopy

SEM was utilized to visualize and confirm the biofilm-forming ability of *A. baumannii* isolates using a Quanta 250 (FEI, Thermo-Fisher Scientific, USA). Biofilm formation was allowed on sterile glass coverslips placed in culture plates and incubated under appropriate conditions. After incubation, the coverslips were carefully removed and gently washed with distilled water or 0.1 M cacodylate buffer for 10-15 minutes to remove non-adherent cells. The adherent biofilm cells on the coverslips were then fixed with 2.5% glutaraldehyde prepared in 0.1 M cacodylate buffer (pH 7.2) and kept at 4°C for 24 hours to ensure proper fixation.

A graded ethanol series (30%, 50%, 70%, 90%, and 100%) was used for stepwise dehydration of the samples, with each step performed for 10-15 minutes. The samples were then air-dried for at least 24 hours. The samples were examined using SEM at an accelerating voltage of 15 kV to assess biofilm structure and architecture. All experiments were conducted in triplicate to ensure reproducibility.

Microscopic observations were performed at multiple magnifications (500×, 600×, 800×, 1,000×, 1,500×, 2,400×, and 3,000×). Corresponding scale bars ranged from 10 µm (higher magnifications) to 200 µm (lower magnifications), enabling accurate visualization of biofilm morphology. Finally, the microscopy images were used to analyze the features of the biofilm, such as the bacterial cell aggregation, surface adhesion, and EPS matrix.

Statistical analysis

Considering TCP as the reference standard, sensitivity, specificity, positive predictive value (PPV), negative predictive value (NPV), and accuracy of CRA were calculated. Correlation between biofilm association genes and antimicrobial resistance was also performed. Associations between biofilm gene presence and phenotypic biofilm production were analyzed using Fisher’s exact test. A p-value <0.05 was considered statistically significant. Analyses were performed using Graphpad Prism 5 software.

## Results

A total of 153 clinical isolates of *A. baumannii* were included in the study (Table 3). Among the patients, females constituted the majority (58.8%, n = 90), while males accounted for 41.2% (n = 63). The mean age of the patients was 49.2 ± 26.31 years, with a wide age range from 10 days to 75 years. Regarding the distribution of respiratory samples, the majority of isolates were obtained from endotracheal aspirates (59.4%, n = 91), followed by tracheostomy tube samples (18.9%, n = 29), sputum (12.4%, n = 19), and bronchial aspirates (9.1%, n = 14). Overall, endotracheal aspirates were the predominant source of *A. baumannii* isolates in this study.

**Table 3:** Clinical isolates of Acinetobacter baumannii from respiratory samples

Parameter	Value (n)	Percentage (%)
Total participants (n = 153)		
Male	63	41.2
Female	90	58.8
Age	49.2 ± 26.31 (10 days to 75 years)	
Endotracheal tube	91	59.4
Tracheostomy tube	29	18.9
Sputum	19	12.4
Bronchial aspirate	14	9.1

Characteristics of the isolates

All 153 clinical isolates were characterized by standard methods used in clinical microbiology and confirmed by biochemical tests. These isolates were Gram-negative coccobacilli that were strictly aerobic, non-fermentative, oxidase-negative, catalase-positive, and non-motile bacteria. They formed mucoid colonies on a Mueller-Hinton agar (MHA) plate.

AST was performed on 12 antibiotics on MHA as per the 2022 CLSI guidelines using the Kirby-Bauer disk diffusion method. The results of 153 isolates for β-lactam/β-lactamase inhibitor, cephalosporin, carbapenem, aminoglycoside, and fluoroquinolone group of drugs are shown in Table 4.

**Table 4:** Antibiotic susceptibility profile of Acinetobacter baumannii isolates.

Class	Antimicrobial drug	Resistant (%)	Intermediate (%)	Sensitive (%)
Aminoglycoside	Amikacin	139 (90.8%)	-	14 (9.1%)
	Gentamicin	134 (87.5%)	-	19 (12.4%)
	Tobramycin	133 (86.9%)	1 (0.65%)	19 (12.4%)
Cephalosporins	Cefepime	145 (94.7%)	-	8 (5.2%)
	Ceftriaxone	148 (96.7%)	0	5 (3.2%)
Sulfonamides	Co-trimoxazole	134 (87.5%)	0	19 (12.4%)
Penicillins + β-lactamase inhibitor	Piperacillin/Tazobactam	141 (92.1%)	11 (7.1%)	1 (0.65%)
Fluoroquinolones	Levofloxacin	136 (88%)	2 (1.3%)	15 (9.8%)
	Ciprofloxacin	148 (96.7%)	0	5 (3.2%)
Carbapenems	Imipenem	146 (95%)	3 (1.9%)	4 (2.6%)
	Meropenem	152 (99%)	-	1 (0.65%)
Tetracyclines	Tetracycline	110 (71%)	17 (11%)	26 (16.9%)

The antimicrobial susceptibility profile demonstrated a high level of resistance among *A. baumannii* isolates. Carbapenems showed the highest resistance, with meropenem (152, 99%) and imipenem (146, 95%), followed by cephalosporins, including ceftriaxone (148, 96.7%) and cefepime (145, 94.7%). High resistance was also observed in fluoroquinolones, with ciprofloxacin (148, 96.7%) and levofloxacin (136, 88%), as well as in the β-lactam/β-lactamase inhibitor combination piperacillin/tazobactam (141, 92.1%). Aminoglycosides also showed high resistance, including amikacin (139, 90.8%), gentamicin (134, 87.5%), and tobramycin (133, 86.9%), along with sulfonamides such as co-trimoxazole (134, 87.5%). In contrast, tetracycline showed comparatively lower resistance (110, 71%).

The MIC distribution of colistin among 153 isolates revealed that 20 (13.1%) isolates had an MIC of 4 µg/mL, whereas the majority (133, 86.9%) of isolates demonstrated an MIC of 1 µg/mL. The calculated MIC₅₀ and MIC₉₀ values were 1 µg/mL and 4 µg/mL, respectively. Many isolates (86.9%) were susceptible to colistin, whereas 13.1% were categorized as resistant, according to the CLSI guidelines.

**Figure 1:**
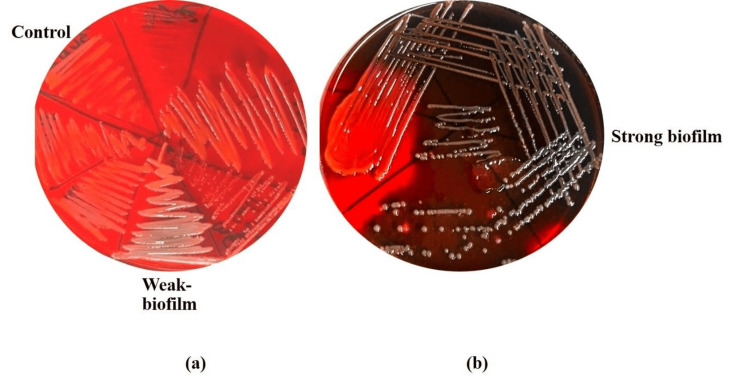
Phenotypic detection of biofilm production using the Congo red agar method. Congo red agar assay for phenotypic detection of biofilm formation. (a) Negative control and weak biofilm-producing isolate exhibiting minimal to moderate black pigmentation. (b) Strong biofilm-producing isolate forming thick black colonies, indicative of high extracellular matrix production.

Out of 153 *A. baumannii* isolates, 57 (37%) were positive for biofilm production on CRA, as indicated by black pigment formation. Among these, 20 (35.1%) isolates were strong biofilm producers, characterized by uniformly black colonies. The remaining 37 (64.9%) isolates were weak biofilm producers, showing dark brown colonies with comparatively paler margins, indicating moderate EPS production. The remaining 96 (62%) isolates retained the original brick-red coloration of the medium and were classified as non-biofilm producers (Figure 1).

The strength of biofilm formation of each isolate was assessed using the TCP method. Table 5 classifies the categories of biofilm formation by the TCP method based on the ability of biofilm formation. The reference strain OD (OD600) was 0.79 for a positive control, *A. baumannii *ATCC19606, and 0.14 for the negative control, *Staphylococcus epidermidis *ATCC12228. In this study, biofilm production was seen in 85 (55.6%) isolates by the TCP method. Among these, 26 (16.9%) isolates were classified as a strong biofilm producer (0D600 > 0.712), 23 (15%) isolates as a moderate biofilm producer (OD600 between 0.356 and 0.712), and 36 (23.5%) as a weak biofilm producer (OD600 between 0.178 and 0.356). Furthermore, 68 (44%) isolates were classified as non-biofilm producers (OD600 < 0.178).

**Table 5:** Categories of biofilm formation using the tissue culture plate method based on the ability of biofilm formation.

Biofilm category	Range of OD600	Number of isolates	Percentage (%)
Strong biofilm producer	>0.712	26	16.9%
Moderate biofilm producer	0.356–0.712	23	15%
Weak biofilm producer	0.178–0.356	36	23.5%
Non-biofilm producer	<0.178	68	44.4%
Total biofilm producer		85	55.6%

Overall, significance was observed between the CRA and TCP methods for biofilm formation (Fisher’s exact test, two-sided p = 0.0433), as shown in Table 6. The calculated odds ratio (OR) was 2.085 (95% confidence interval (CI) = 1.055-4.119), with a PPV of 66.7%, an NPV of 51%, and an accuracy of 56.86%.

**Table 6:** Diagnostic performance of CRA compared with tissue culture plate (TCP) assay for the phenotypic detection of biofilm formation in Acinetobacter baumannii. CRA = Congo red agar; TCP = tissue culture plate; PPV = positive predictive value; NPV = negative predictive value; CI = confidence interval

TCP assay (reference standard)	CRA positive	CRA negative	Diagnostic parameters (95% CI)				
Biofilm producers (n = 85)	38 (24.8%)	47 (30.7%)	Sensitivity	Specificity	PPV	NPV	Accuracy
Non-biofilm producers (n = 68)	19 (12.4%)	49 (32.0%)	44.7%	72.1%	66.7%	51.0%	56.9%

**Figure 2:**
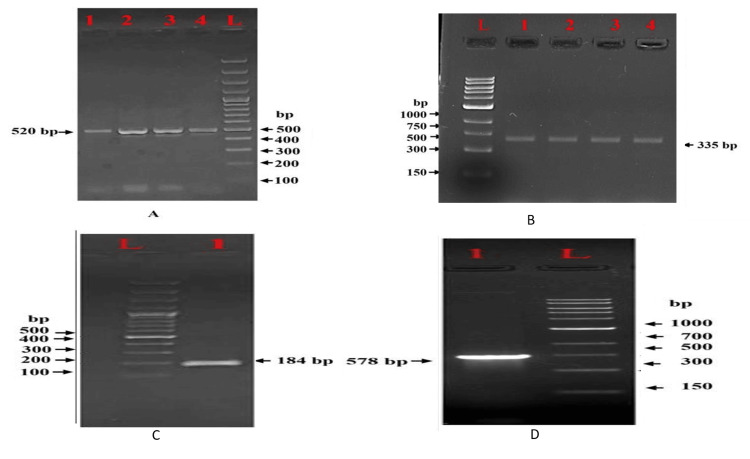
Representative PCR gel (2%) agarose showing the biofilm-producing gene in this study. Each band represents the amplicon of one of the positive isolates for given with 100 bp/1,000 bp ladder (L). (A) Lanes 1-4 show *blaPER-1* (520 bp) L-100 bp. (B) L-150 bp/1,000 bp ladder lanes 1-4 *csuD* (335 bp). (C) ladder 100 bp/1,000 bp, lane 1 *bap* (184 bp). (D) Lane 1 *ompA* (578 bp) and L-150 bp/1,000 bp ladder. PCR = polymerase chain reaction

In this study, 153 *A. baumannii* isolates were evaluated for biofilm-related genes. The result showed that *csuD* was the most prevalent gene, detected in 99 (64.7%) isolates, followed by *bap *(82, 53.6%), *blaPER-1 *(23, 15%), and *ompA *(8, 5.2%) (Figure 2).

**Figure 3:**
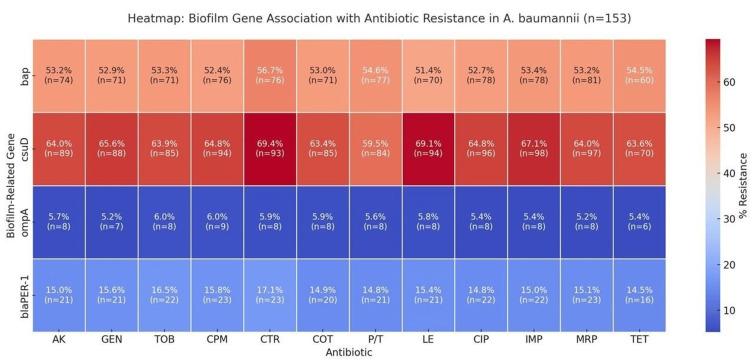
Heatmap illustrating the association between biofilm-related genes (bap, csuD, ompA, blaPER-1) and resistance to 12 antibiotics among clinical Acinetobacter baumannii isolates (n = 153). Each cell displays the percentage of resistant isolates harboring a specific gene and the corresponding number of strains (n). Notably, *csuD* showed the highest association with multiple drug resistance followed by bap gene. The presence of *ompA* was minimal across all drugs. AK = amikacin; GEN = gentamicin; TOB = tobramycin; CPM = cefepime; CTR = ceftriaxone; COT = cotrimoxazole; P/T = piperacillin-tazobactam; LE = levofloxacin; CIP = ciprofloxacin; IMP = imipenem; MRP = meropenem; TE = tetracycline

The heat map shown in Figure 3 depicts the distribution of four key biofilm-associated genes, namely,* bap*,* csuD*,* ompA*,* and blaPER-1,* across isolates resistant to 12 antibiotics. Each cell shows the percentage of resistant isolates harboring the gene and the actual strain count.

The findings of the biofilm-associated genes, especially *csuD*, are more strongly associated with all MDR levels (64-69%), particularly against ceftriaxone (93, 69.4%), levofloxacin (94, 69.1%), imipenem (98, 67.1%), and meropenem (97, 64%), indicating a predominant role in biofilm-mediated resistance in the study population. In contrast, the *bap* gene against ceftriaxone (76, 56.7%) and piperacillin/tazobactam (77, 54.6%) showed only a non-significant finding, indicating a weak contribution. However, these findings indicate that within this study cohort, *csuD* was more strongly associated with antibiotic resistance and had a significant role in biofilm-mediated resistance. *blaPER-1*, although less prevalent than *csuD or bap*, maintained a steady presence (~15-17%) across most drugs. *ompA* occurred at very low levels (5-6%) irrespective of the antibiotic, indicating a minor contribution to resistance in this cohort.

The association between the combined presence of *bap* and *csuD* genes and biofilm formation was analyzed using Fisher’s exact test, as presented in Table 7. Among the 153 *A. baumannii *isolates, 50 were positive for both *bap* and *csuD*, of which 34 (68.0%) were biofilm producers and 16 (32.0%) were non-producers. In contrast, among 103 isolates lacking this gene combination, 51 (49.5%) were biofilm producers and 52 (50.5%) were non-producers.

**Table 7:** Association of biofilm-associated genes (bap, ompA, csuD, blaPER-1) with biofilm strength as determined by the TCP assay among Acinetobacter baumannii isolates (n = 153). The most common gene combination of strong biofilm production was *bap* + *csuD* (p < 0.037). TCP = tissue culture plate

Gene(s) detected	Total isolates n (%)	Non-producers (n = 68)	Weak producers (n = 36)	Moderate producers (n = 23)	Strong producers (n = 26)	Total biofilm producers (n = 85)	P-value
Single gene							
csuD	99 (64.7)	42 (42.4)	24 (24.2)	15 (15.2)	18 (18.2)	57 (57.6)	0.13
Bap	82 (53.6)	32 (39.0)	21 (25.6)	13 (15.8)	16 (19.5)	50 (60.9)	0.19
blaPER-1	23 (15.0)	8 (34.8)	7 (30.4)	4 (17.4)	4 (17.4)	15 (65.2)	0.14
ompA	8 (5.2)	5 (62.5)	3 (37.5)	–	–	3 (37.5)	0.40
Two-gene combinations							
bap + csuD	51 (33.3)	17 (33.3)	16 (31.4)	7 (13.7)	11 (21.6)	34 (66.7)	0.037*
bap + blaPER-1	13 (8.5)	4 (30.8)	3 (23.1)	3 (23.1)	3 (23.1)	9 (69.2)	0.16
csuD + blaPER-1	13 (8.5)	6 (46.2)	4 (30.8)	1 (7.7)	2 (15.4)	7 (53.8)	0.70
ompA + csuD	4 (2.6)	3 (75.0)	1 (25.0)	–	–	1 (25.0)	0.17
ompA + blaPER-1	2 (1.3)	1 (50.0)	1 (50.0)	–	–	1 (50.0)	0.50
Three-gene combinations							
bap + csuD + blaPER-1	6 (3.9)	2 (33.3)	2 (33.3)	1 (16.7)	1 (16.7)	4 (66.7)	0.50
bap + ompA + csuD	3 (2.0)	2 (66.7)	1 (33.3)	–	–	1 (33.3)	0.50
bap + ompA + blaPER-1	1 (0.7)	1 (100)	–	–	–	–	–
Four-gene combination							
bap + ompA + csuD + blaPER-1	1 (0.7)	1 (100)	–	–	–	–	0.43

**Figure 4:**
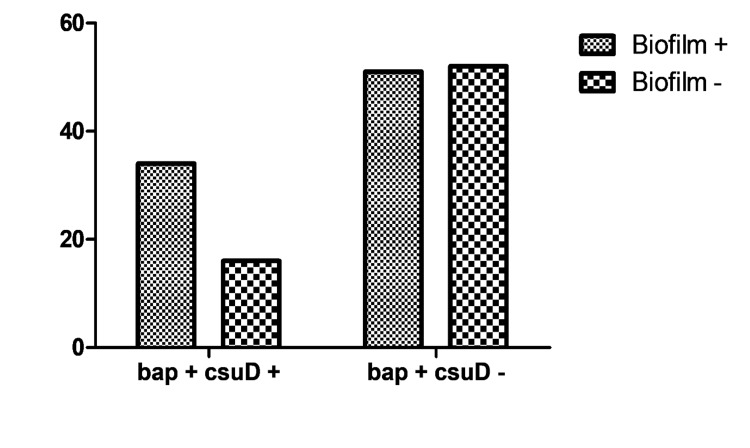
Odds ratio with 95% confidence intervals, calculated using Fisher’s exact test, to evaluate the strength of association between biofilm-associated genes and biofilm formation as determined by the TCP method. X axis represents gene combination and Y axis represents the number of isolates. TCP = tissue culture plate

Statistical analysis demonstrated a significant association between the co-presence of *bap* and *csuD* genes and biofilm formation (p = 0.037), as illustrated in Figure 4. The OR was 2.167 (95% CI = 1.066-4.402), indicating that isolates harboring both genes were approximately 2.1 times more likely to form biofilms compared to those without this combination. Similarly, the relative risk was 1.373 (95% CI = 1.046-1.803), suggesting a higher probability of biofilm formation in isolates with both genes.

Additionally, the most common gene combinations were *bap + blaPER-1 *(n = 13, 8.5%) and *csuD + blaPER-1 *(n = 13, 8.5%). The combination of *omp + blaPER-1* was present in only two isolates (1.3%).

Ultrastructural confirmation of biofilm architecture by scanning electron microscopy

**Figure 5:**
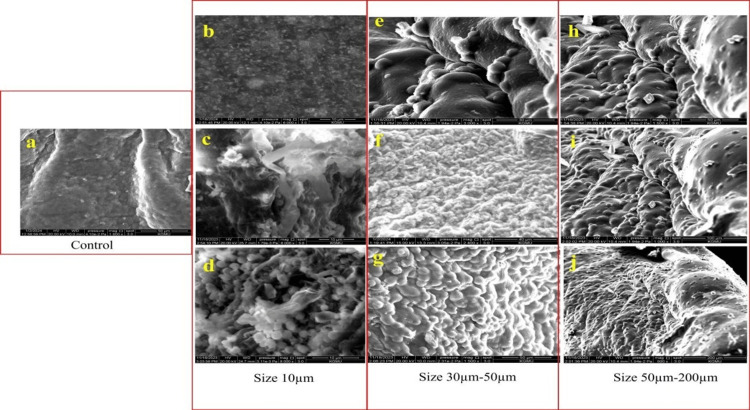
Biofilm morphology as a strong biofilm harboring the biofilm gene, along with positive and negative controls on the glass coverslip surface visualized by scanning electron microscopy with higher to lower magnification (a) A non-biofilm-forming isolate. (b-d) Biofilm architecture at magnifications of 6,000×, 600×, and 800× with a scale bar of 10 µm. (e-g) Biofilm structures at 3,000×, 2,400×, and 1,500× with scale ranges of 30-50 µm. (h-j) Lower magnification images at 1,500×, 1,000×, and 500×, corresponding to scale ranges of 50-200 µm, demonstrating the overall biofilm organization and surface coverage.

A total of 20 isolates identified as strong biofilm producers classified by the TCP method and harboring the biofilm genes of *csuD* and *bap*, along with positive and negative controls, were visualized by SEM. Biofilms were developed on a glass cover-slip surface with different magnifications and scale ranges (Figure 5). The negative control (non-biofilm-forming isolate) appeared smooth and uncolonized, with no detectable extracellular polymeric matrix at higher magnification to lower magnification (10 µm, 30-50 µm, and 50-200 µm) (Figure 5a). Figures 5c-5d illustrate the initial stages of biofilm development, characterized by early bacterial adhesion and the onset of EPS production. This stage plays a crucial role in surface attachment and the initiation of biofilm formation. The presence of matrix material and channel-like structures within the EPS suggests the beginning of nutrient diffusion pathways. Figures 5e-5g) demonstrate more advanced biofilm development, showing densely packed bacterial communities embedded within a well-defined EPS matrix. Predominantly, coccobacillary cells were observed, and the matrix acted as a scaffold that promoted intercellular adhesion and structural organization, facilitating stable biofilm formation. In Figures 5h-5j, a mature biofilm architecture is evident, displaying highly organized, multilayered structures with bridge-like connections between adjacent microcolonies. These images reveal a three-dimensional, heterogeneous biofilm with an irregular EPS network, contributing to enhanced mechanical stability and integrity of the biofilm.

**Figure 6:**
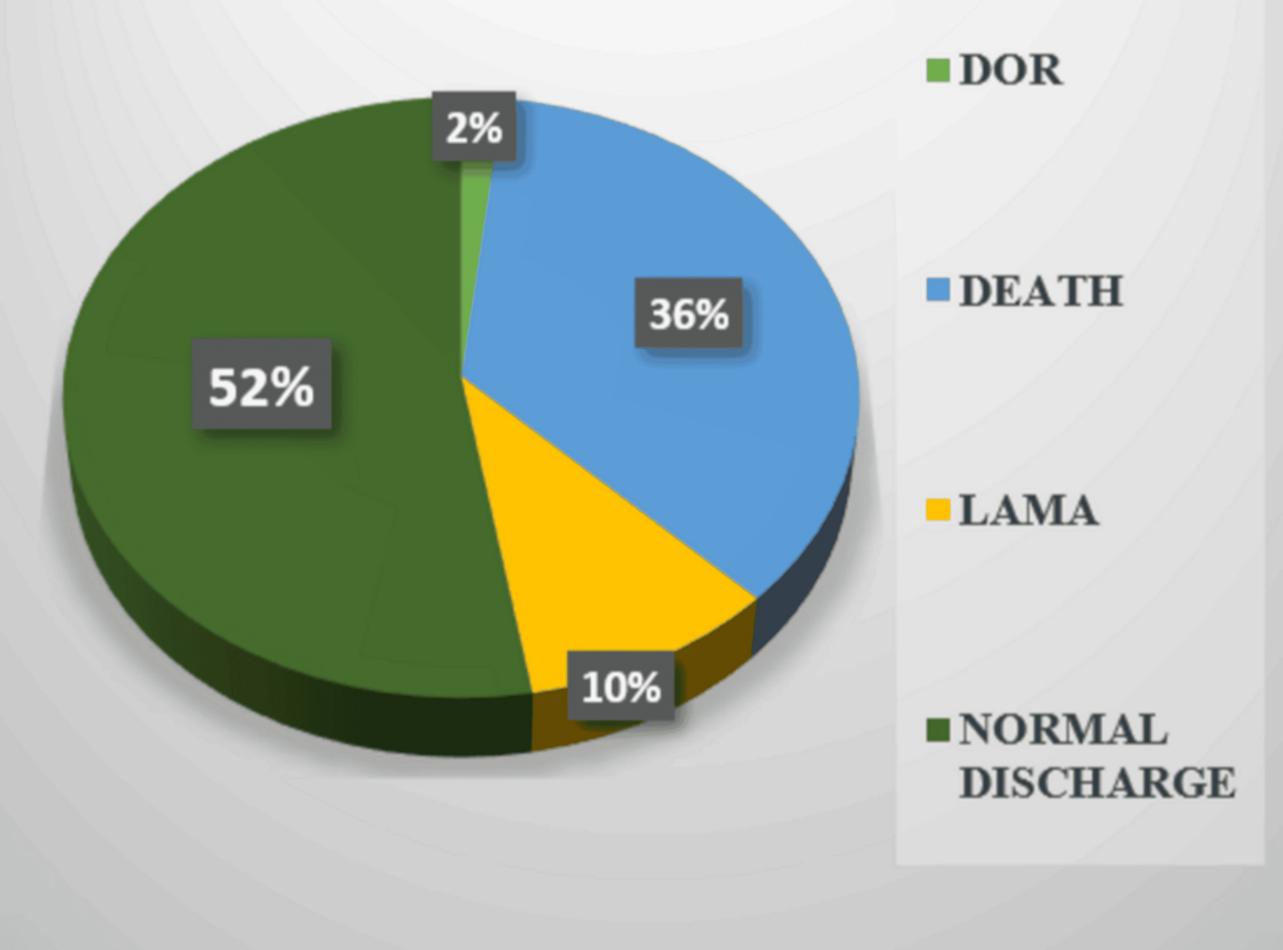
Pie chart showing patient outcomes. LAMA = left against medical advice; DOR = discharged on request

Figure 6 illustrates the patient outcome observed in this study population. Among the cases, 52% of patients were discharged under normal conditions, while 36% resulted in mortality. Additionally, 10% of patients left against medical advice, and 2% were discharged on request. These findings highlight a considerable mortality rate and emphasize the urgent need for strengthened infection control strategies and optimized therapeutic interventions to improve clinical outcomes and reduce patient deaths.

## Discussion

Biofilms are organized bacterial colonies that are shielded from environmental stressors and antimicrobial drugs by the self-production of extracellular polymeric matrix materials. Biofilm formation plays a significant role in bacterial pathogenicity and makes the treatment of infections difficult. In the present study, 153 *A. baumannii *isolates collected from respiratory specimens were evaluated for their biofilm-forming ability using both phenotypic and genotypic approaches.

In the present study, strong biofilm formation was identified in 20 (13.07%) isolates by the CRA method and in 26 (16.99%) isolates by the TCP method, indicating a slightly higher detection rate with the quantitative TCP assay. These findings are consistent with previous studies reporting variability between the two methods. For instance, some studies have reported lower detection rates, with 7.5% (CRA) and 4.2% (TCP), while others observed 10.3% and 8.3% biofilm production by CRA and TCP, respectively. In contrast, higher prevalence rates have also been documented, such as 69% (TCP) and 55% (CRA) [11].

The differences observed across studies may be attributed to several factors, including variations in sample type (e.g., respiratory versus other clinical specimens), differences in methodological protocols such as media composition, incubation conditions, and cut-off values for biofilm classification, as well as inherent strain variability among *A. baumannii* isolates. Additionally, the TCP method, being a quantitative assay, is generally considered more sensitive and reliable compared to the qualitative CRA method, which relies on subjective interpretation of colony morphology. In our study, the accuracy and detection rates of CRA and TCP were comparable to previously reported data, particularly in studies involving respiratory isolates, further supporting the reproducibility of our findings [12]. Similar observations were reported in the increased biofilm formation in endotracheal tubes, which may be attributed to the presence of a foreign body and a favorable microenvironment, including mucus build-up and stagnation, which support biofilm development [13].

In this study, the presence of the *csuD and bap* genes, individually or together, showed the strongest association with phenotypic biofilm formation. A previous study showed similar results for the biofilm-producing gene of *csuD *[14,15]. Another biofilm gene, the *bap *gene, which is widely distributed across different regions, has been reported in previous studies [16-18]. The *bap* gene promotes pili attachment, facilitates the outer cell wall, helps attach to another wall, exchanges the genetic material, produces EPS, and forms biofilms [19]. In contrast to earlier reports, the detection rates of the *ompA* genes in our study were markedly lower. *ompA *was identified in eight (5.2%) isolates. Previous studies, however, have reported much higher prevalence, with *ompA* ranging from 68.8% to 73% [20,21]. *ompA* in *A. baumannii* is known to play a crucial role in adhesion to human epithelial cells, biofilm development, and antimicrobial resistance. In this study, the *blaPER-1 *gene was identified in 23 (15%) isolates, which is consistent with earlier reports noting its presence in 10%, 30%, and 38% of isolates. The *blaPER-1 *is an extended-spectrum lactamase gene that has also been shown to contribute to biofilm formation on surfaces, thereby supporting bacterial persistence.

In addition to evaluating the correlation between biofilm formation and the expression of biofilm-associated genes, this study also assessed the relationship between antimicrobial resistance patterns and biofilm formation. SEM revealed well-organized biofilm architecture in strong biofilm formation, corroborating the intricacy and endurance, which supports the study’s findings. As biofilms can greatly restrict antibiotic penetration and shield bacteria from host immune responses, the confluence of biofilm development with MDR poses a considerable therapeutic challenge from a clinical point of view, resulting in long-term infections, a higher chance of treatment failure, and increased morbidity, especially in critically ill patients.

Furthermore, the high correlation between *csuD and bap*, both separately and together, indicates that both genes are essential for increasing *A. baumannii* virulence, persistence, and resistance. Thus, regular examinations for genes linked to biofilms and phenotypic identification of biofilm formation may help in risk assessment and direct more efficient therapeutic approaches. These results also highlight the need for stronger infection control procedures and alternate therapeutic strategies, such as combination antibiotic therapy and antibiofilm medicines, to stop the spread of these extremely resistant infections. Overall, incorporating biofilm-related factors into clinical judgment may enhance patient outcomes and aid in the fight against *A. baumanni-*related infections linked to healthcare.

Limitations

This study has some limitations. First, it was conducted at a single tertiary care center, limiting generalizability. Second, only a few biofilm-related genes were analyzed. Third, the absence of whole-genome sequencing limited the identification of additional resistance genes and biofilm-related pathways, an important concern in high-burden settings, as highlighted by the Centers for Disease Control and Prevention.

## Conclusions

This study demonstrated a high prevalence of MDR and biofilm formation among *A. baumannii* isolates, with notable resistance to carbapenems and cephalosporins. Heatmap analysis showed that the *csuD* gene was strongly correlated with resistance to ceftriaxone and meropenem. Gene combination analysis further revealed a significant association, where the presence of both the *bap* and *csuD* genes was linked to higher biofilm production. SEM findings supported these results by demonstrating dense and well-structured biofilm architecture in strong biofilm producers. Overall, the study concluded that biofilm formation plays a key role in antimicrobial resistance and persistence of *A. baumannii,* highlighting the need for targeted strategies to control biofilm-associated infections.
